# Deletion in *RMST* lncRNA impairs hypothalamic neuronal development in a human stem cell-based model of Kallmann Syndrome

**DOI:** 10.1038/s41420-024-02074-4

**Published:** 2024-07-19

**Authors:** Gowher Ali, Kyung Chul Shin, Nisar Ahmed, Wesal Habbab, Ghaneya Alkhadairi, Aleem Razzaq, Yosra Bejaoui, Nady El Hajj, Borbala Mifsud, Yongsoo Park, Lawrence W. Stanton

**Affiliations:** 1 Neurological Disorders Research Center, Qatar Biomedical Research Institute, Hamad, Bin Khalifa University, Qatar Foundation, Doha, Qatar; 2 College of Health & Life Sciences, Hamad Bin Khalifa University, Qatar Foundation, Doha, Qatar; 3 College of Science and Engineering, Hamad Bin Khalifa University, Qatar Foundation, Doha, Qatar; 4 William Harvey Research Institute, Queen Mary University London, London, UK

**Keywords:** Stem-cell differentiation, Neural tube defects

## Abstract

Rhabdomyosarcoma 2-associated transcript (*RMST*) long non-coding RNA has previously been shown to cause Kallmann syndrome (KS), a rare genetic disorder characterized by congenital hypogonadotropic hypogonadism (CHH) and olfactory dysfunction. In the present study, we generated large deletions of approximately 41.55 kb in the *RMST* gene in human pluripotent stem cells using CRISPR/Cas9 gene editing. To evaluate the impact of *RMST* deletion, these cells were differentiated into hypothalamic neurons that include 10–15% neurons that express gonadotrophin-releasing hormone (GnRH). We found that deletion in *RMST* did not impair the neurogenesis of GnRH neurons, however, the hypothalamic neurons were electro-physiologically hyperactive and had increased calcium influx activity compared to control. Transcriptomic and epigenetic analyses showed that *RMST* deletion caused altered expression of key genes involved in neuronal development, ion channels, synaptic signaling and cell adhesion. The in vitro generation of these *RMST*-deleted GnRH neurons provides an excellent cell-based model to dissect the molecular mechanism of *RMST* function in Kallmann syndrome and its role in hypothalamic neuronal development.

## Introduction

Kallmann syndrome (KS) is a rare genetic disorder characterized by congenital hypogonadotropic hypogonadism (CHH) and olfactory dysfunction [1–3]. Individuals with KS show symptoms like delayed or absent puberty, infertility, low level of gonadotropin and an altered sense of smell (hyposmia or anosmia). Olfactory dysfunction is related to the absence or hypoplasia of the olfactory bulbs and tract, while hypogonadism is due to gonadotropin-releasing hormone (GnRH) deficiency, presumably resulting from the impaired migration or development of GnRH cells [4, 5]. GnRH precursor cells are born in the olfactory placode and migrate along the olfactory axons to their ultimate destination in the hypothalamus [6–9]. Defects in the neurogenesis of GnRH cells, differentiation or axons extension to hypothalamus median eminence also contribute to KS and other CHH in some genetic forms of the disease. Rare genetic variations in more than 30 different genes have been implicated in KS, affecting GnRH neuronal development and migration [3, 10–13]. A recent study in one KS proband identified a balanced t(7;12)(q22;q24) chromosomal translocation disrupting *RMST*, a long non-coding RNA (lncRNA) gene on Chr12 not previously linked to CHH [14].

lncRNAs are abundant in the mammalian transcriptome and recent studies show that lncRNAs are involved in homeostasis and function of the mammalian brain as well as in the pathophysiology of brain-related diseases including neurodevelopmental disorders [15–18]. One such example is *RMST* lncRNA, which was identified as a regulator of neurogenesis in a genome-wide screen using neurons generated by directed differentiation of human embryonic stem cells (hESC), and knock-down of *RMST* by RNA interference blocked neurogenesis in vitro [19]. *RMST* expression is specific to brain and play an essential role during dopaminergic neuronal differentiation [20]. Overexpression of *RMST* is identified in oxygen-glucose deprived HT-22 hippocampal neuron cell line and silencing *RMST* significantly inhibited neuronal apoptosis [21]. *RMST* was found to localize in the nucleus and physically interact with the SOX2, a transcription factor regulating neural cell fate, and was required for the binding of SOX2 to promoter regions of neurogenic transcription factors to co-regulate hundreds of target genes implicated in neurogenesis [22].

Given the importance of *RMST* in regulating neurogenesis, we hypothesized that the balanced t(7;12)(q22;q24) chromosomal translocation in the KS proband resulted in a loss of *RMST* function, thereby leading to impaired function or development of hypothalamic neurons and the clinical manifestations observed. We tested this hypothesis by generating *RMST*-deleted hPSCs lines using CRISPR/Cas9 gene editing. The data presented here indicates that *RMST*-deletion does not affect the generation of GnRH neurons, however, the neurons were electro-physiologically hyperactive and have increased calcium influx activity compared to control. Moreover, transcriptome and epigenetic analyses show that *RMST* deletion caused altered expression of key genes involved in the neuronal development, ion channels, synaptic signaling and cell adhesion. These hPSCs-derived *RMST*-deleted GnRH neurons provide an excellent cell-based model to further understand the molecular mechanism of *RMST* lncRNA during GnRH neurons differentiation and in the progression of Kallmann syndrome.

## Results

### CRISPR-Cas9 mediated deletion of *RMST* in human pluripotent stem cells (hPSCs)

**Fig. 1:**
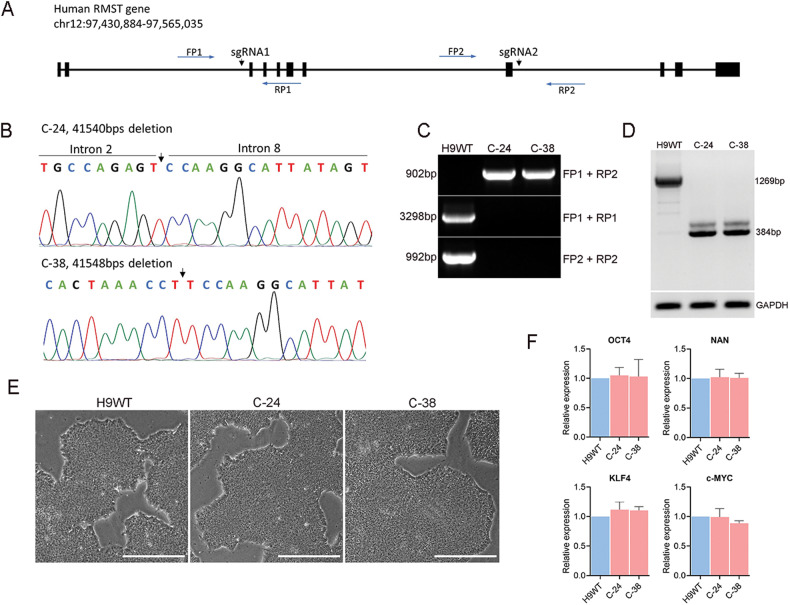
Genomic deletion of *RMST* in hPSCs. **A** Schematic illustration of *RMST* gene locus (GRCh38/hg38, chr12:97,430,884-97,565,035) and targeting strategy for CRISPR-Cas9 mediated editing. *RMST* exons are indicated by black rectangles and black arrows indicate single guide RNA (sgRNA) targeting sites. sgRNA1 and sgRNA2 (Table S1) targeted upstream of exon 3 and downstream of exon 8, respectively. Forward primers (FP1 and FP2) and reverse primers (RP1 and RP2) shown in blue arrows were used for genotyping and sequencing. Primer sequences are listed in Table S1. **B** Sanger sequencing of PCR products in single cell-derived clones showing deletion in *RMST* gene following CRISPR-Cas9 editing. Clone-24 (C-24) has a 41.540 kb deletion and clone-38 (C-38) has a 41.548 kb deletion. **C** Agarose gel electrophoresis of PCR-amplified genomic DNA for the validation of genetic deletion of *RMST* with indicated forward primers (FP1 and FP2) and reverse primers (RP1 and RP2). **D** RT-PCR of RMST cDNA in hPSCs. In H9WT cell, *RMST* transcript amplification yielded an amplicon length of 1269 bp with the primers listed in Table S2, whereas *RMST* transcript is truncated in C-24 and C-38 clones yielding an amplicon of 384 bp. **E** Bright field morphology of H9WT, C-24, and C-38 hPSCs. Scale, 100 µm. **F** qPCR for the validation of pluripotency markers *OCT4, NAN, SOX2, KLF4* and *C-MYC* in *RMST*-deleted hPSCs.

We hypothesized that loss of *RMST* function leads to impaired function or development of hypothalamic neurons. To test this hypothesis, we sought to generate hypothalamic neurons from *RMST*-deleted hPSCs. To this end we used CRISPR-Cas9 genome editing to delete *RMST* in H9 wild-type (H9WT) hPSCs (WA09, WiCell Research Institute). A pair of sgRNAs targeted *RMST* upstream of exon 3 and downstream exon 8 (Fig. 1A). Nonhomologous end-joining repair of the two double-strand breaks created by sgRNA-targeted Cas9 were expected to introduce a major deletion in the *RMST* gene. After nucleofection and single cell plating, multiple clones were obtained with heterozygous or homozygous deletion (Fig. S1A). Two clones, named clone-24 and clone-38 (C-24 and C-38), were further characterized as having homozygous deletion of 41,540 bp and 41,548 bp respectively. Deletion in *RMST* was confirmed by DNA sequencing (Fig. 1B) and gel electrophoresis of PCR-amplified genomic DNA (Fig. 1C). Primer pair (FP1 and RP2) amplify the entire region spanning exon 3 to exon 8 (Fig. 1A) consisting of 42,802 bp, and such a large sequence prevents PCR amplification from wild-type genomes. However, in *RMST*-deleted clones, intron 2 and intron 8 are conjoined such that the primer sites come into proximity yielding a 902 bp amplicon (Fig. 1B, C). The use of primer pairs (FP1 and RP1) and (FP2 and RP2) resulted in expected amplification products in wild-type hPSCs, whereas in clones C-24 and C-38 which lack binding sites for FP2 and RP1 primers after deletion, no PCR amplification was observed (Fig. 1C). These results confirmed the homozygous deletion of the *RMST* gene in C-24 and C-38 hPSCs.

We assessed the expression of *RMST* transcripts in C-24 and C-38 and wild-type cells. Deletion of *RMST* was confirmed by RT-PCR using the forward primer binding to exon 1 and exon 2 junction and reverse primer binding to exon 10 (listed in Table S2). The PCR amplification of *RMST* cDNA in wild-type hPSCs resulted in an amplicon of 1269 bp, whereas in C-24 and C-38, the *RMST* transcript was truncated as indicated by an amplicon of 384 bp (Fig. 1D). This result indicates that genomic deletion in *RMST* results in expression of substantially truncated transcripts.

The C-24 and C-38 hPSCs lines showed typical stem cell morphology indistinguishable from wild type (Fig. 1E) and normal karyotype (Fig. S1B). We also found that pluripotency-related genes *OCT4*, *NANOG*, *KLF4,* and *C-MYC* were equivalently expressed in all the lines (Fig. 1F), indicating that deletion in *RMST* is dispensable for maintaining hPSCs pluripotency. In conclusion, we demonstrated successful generation of stable hPSCs lines harboring large genomic deletions in the *RMST* gene.

### Directed differentiation of hPSCs into GnRH neurons

**Fig. 2:**
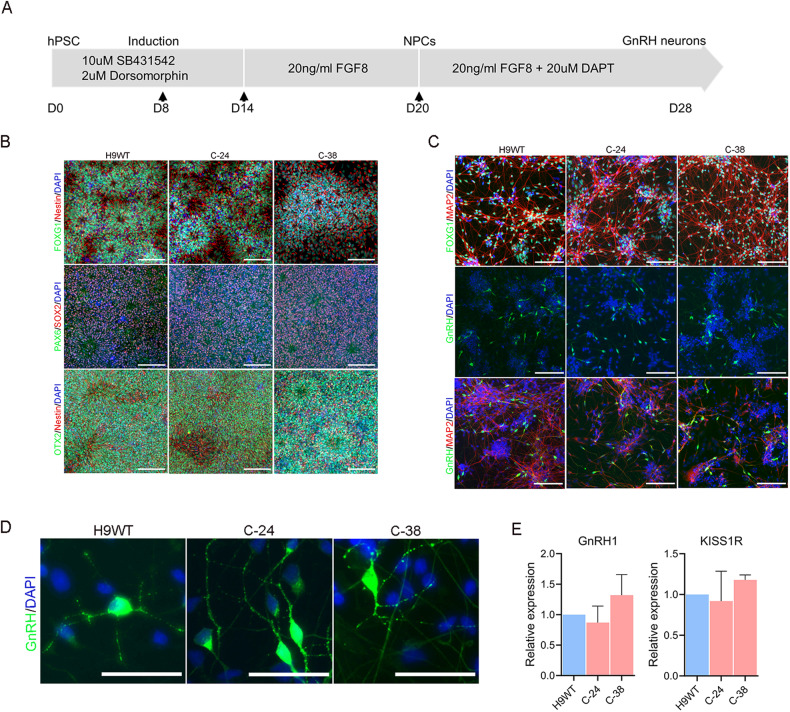
Differentiation of hPSCs into hypothalamic GnRH neurons. **A** Schematic representation of the protocol depicting the stepwise differentiation of hPSCs into GnRH neurons. For the first 12 days, cells were treated with dual SMAD inhibitors, SB431442 and Dorsomorphin. At day 12 of differentiation, FGF8 was added and from day 21 onwards, FGF8 and notch inhibitor DAPT were added to the culture medium. Arrows indicate the time of cell splitting. **B** Immunostaining of cells on day 20 of differentiation showing the expression of progenitor markers SOX2, PAX6, OTX2, FOXG1, and Nestin. **C** Immunostaining of mature GnRH neurons showing the expression of MAP2, GnRH, and FOXG1 on day 28 of differentiation. **D** High-resolution images of GnRH neurons showing the expression and punctuate staining of GnRH. Arrows indicate the GnRH containing vesicles. Cell nuclei were stained with DAPI (blue). **E** qPCR of GnRH1 and *KISS1R* in differentiated GnRH neurons. Graphs show mean with ±SEM of 3–4 independent biological replicates and the data were analyzed using unpaired student *t*-test. Primer sequences are listed in Table S2. **B**, **C** Scale, 100 µm. **D** Scale, 50 µm.

Next, we generated hypothalamic neurons that included GnRH-expressing neurons from wild-type and *RMST*-deleted hPSCs using a published protocol [23, 24]. A schematic illustration of hPSCs differentiation into GnRH neurons is presented in Fig. 2A. For GnRH neurons differentiation, hPSCs were treated for 12 days with dual SMAD inhibitors SB431542 and dorsomorphin to block TGF-b/activin and BMP signaling pathways, respectively [25]. This was followed by treatment with FGF8, which functions as a key growth factor in the development of GnRH neurons. On day 20 of differentiation, the cells organized in neuronal rosettes and immunostaining confirmed the homogenous expression of neuroectodermal markers SOX2, PAX6 and NESTIN, indicating efficient neural conversion (Fig. 2B). The anterior fate of the cells was confirmed by staining with FOXG1 and OTX2 (Fig. 2B). No differences in the expression of progenitor markers were observed in control and *RMST*-deleted hPSCs-derived NPCs using RT-qPCR (Fig. S2A).

To induce differentiation of NPCs into GnRH neurons, cells were treated with FGF8 and Notch inhibitor DAPT for one week. The neuronal identity of differentiated cells was confirmed by immunostaining with MAP2 and FOXG1 (Fig. 2C). Immunofluorescence showed the presence of GnRH-positive cells and most of the cells were expressing FOXG1 (Fig. 2C). The number of proliferating (Ki67 positive) cells was very low, indicating that the cells were terminally differentiated and exited the cell cycle (Fig. S2B). High-magnification images of GnRH-expressing cells showed a punctate staining pattern in most cells, indicating vesicular packaging of GnRH decapeptide (Fig. 2D). The neurons express kisspeptin receptor (KISS1R) which binds kisspeptin, a neuropeptide triggering the release of GnRH and there were no significant differences in GnRH and KISS1R transcripts level among the lines (Fig. 2E). In conclusion, hPSCs were efficiently differentiated into mature GnRH neurons and no significant differences in differentiation potential were observed between the control and *RMST*-deleted cells.

### Physiological characterization of hPSCs-derived GnRH neurons

**Fig. 3:**
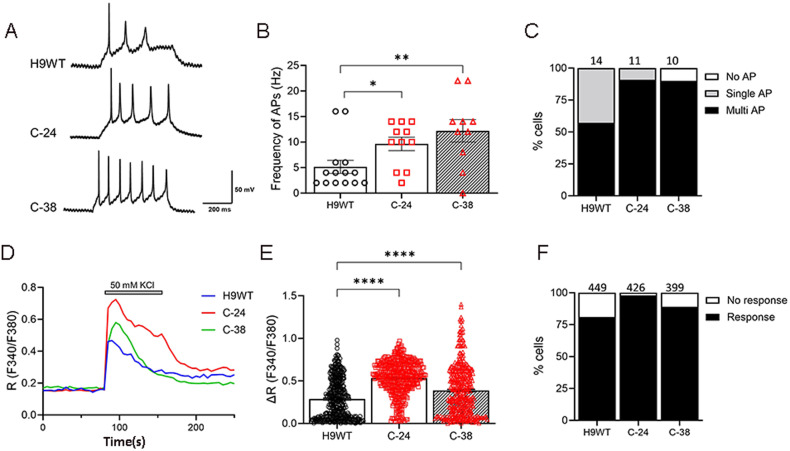
Electrophysiological characterization of *RMST*-deleted GnRH neurons. **A** Whole-cell patch-clamp recording to monitor action potential (AP) generated by injection of current pulses in a current-clamp mode; no AP, single AP, or multiple and repetitive AP. **B** AP frequency of control and mutant neurons after one week differentiation. Data are means ± SEM from 3 independent differentiations: *n* = 14 (control), 11 (C-24), 10 (C-38). One-way ANOVA with Tukey’s multiple comparisons was used. *, *p* < 0.05. **p < 0.01. **C** Distributions of AP generation; no AP, single AP, or multiple/repetitive AP in neurons differentiated for one week. **D** Representative traces of intracellular calcium ions (Fura-2 F340/F380 ratio) in neurons stimulated by 50 mM KCl. **E** Net changes of calcium increase by 50 mM KCl. Data are means ± SEM from 3 independent differentiation: *n* = 449 (control), 426 (C-24), 399 (C-38). One-way ANOVA with Tukey’s multiple comparisons was used. ****p < 0.0001. **F** Percentage of neurons that evoke calcium influx by 50 mM KCl. The number of cells tested are shown from 3 independent differentiation.

We investigated the functional properties of *RMST*-deleted neurons by measuring their action potentials (AP) and calcium influx. We used whole-cell patch-clamp recordings to assess the AP firing patterns of in vitro-generated neurons. We found that both control (H9WT) and mutant neurons (C-24, C-38) generated multiple and repetitive AP (Fig. 3A, C). Notably, deletion in *RMST* significantly increased the AP frequency compared to control hPSCs-derived neurons (Fig. 3B). In addition, the proportion of neurons that generated multiple AP was higher in mutant neurons than in control (57%); C-24 (90.9%), C-38 (90%) (Fig. 3C).

Single-cell calcium imaging was performed to further assess the functional activity of *RMST*-deleted neurons (Fig. 3D, F). Calcium influx through voltage-gated calcium ion channels (VGCC) is essential for synaptic transmission and plasticity. We stimulated neurons with 50 mM KCl and measured the calcium influx through VGCC. We observed that *RMST* deletion enhanced the calcium influx (Fig. 3E), and the percentage of neurons that responded to KCl also increased (Fig. 3F); control (81%), C-24 (98%), and C-38 (89%). These results suggest that neurons derived from *RMST*-deleted cells are functionally mature and electro-physiologically more active than control neurons.

### Gene expression analysis of GnRH neurons

**Fig. 4:**
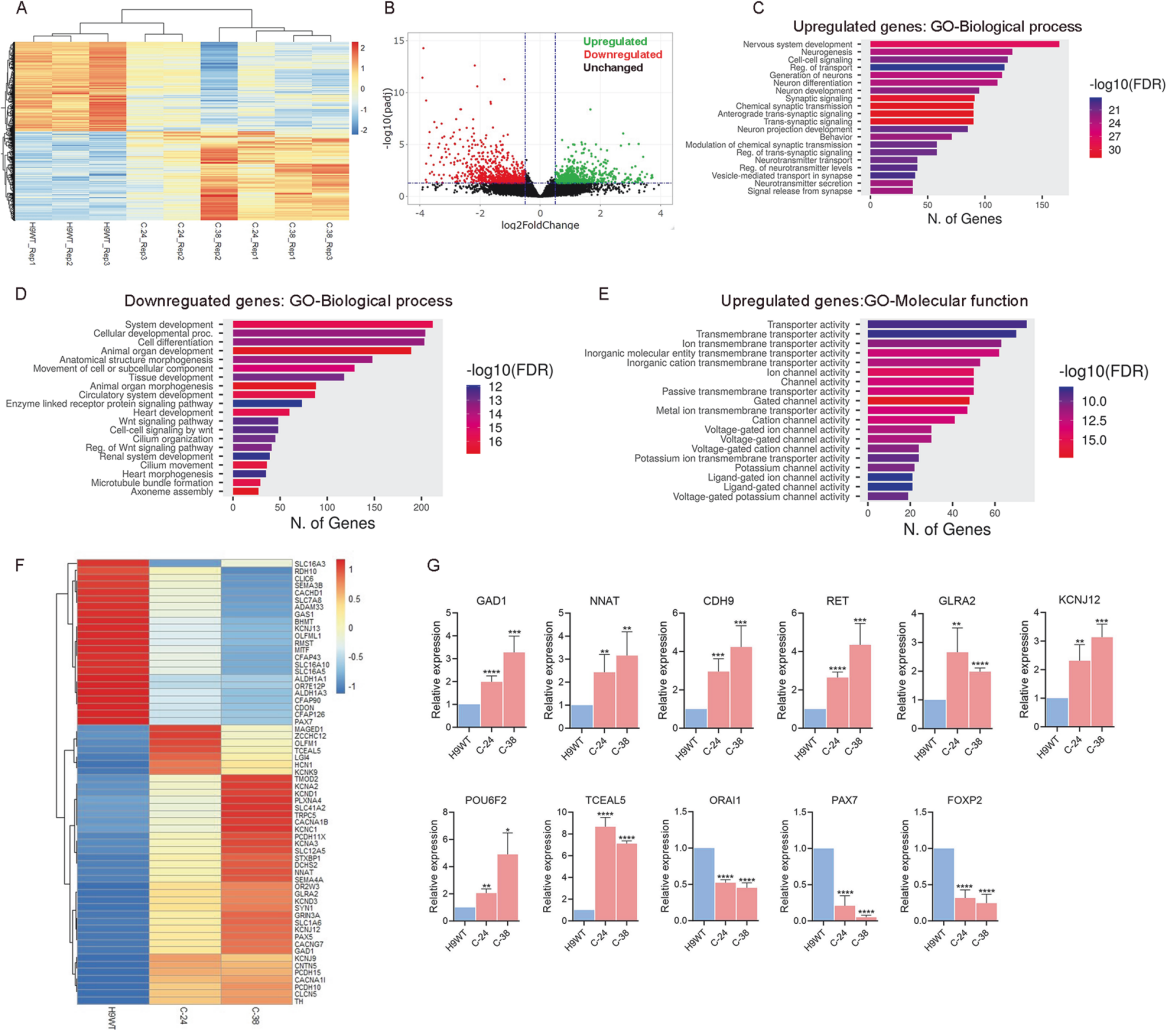
RNA-seq analysis of hPSCs-derived hypothalamic GnRH neurons. **A** Heatmap of all differentially expressed genes (DEGs) in *RMST*-deleted hPSCs-derived GnRH neurons compare to control H9WT. Three independent biological replicates from each sample were analyzed (*P*-value < 0.05 and 1.5-fold change). Expression data have been standardized as z-scores for each mRNA. **B** Volcano plot showing the log2 fold change and the adjusted *P*-value for all the detected transcripts; upregulated (green), downregulated (red), unchanged (black). **C**, **D** Gene ontology (GO) enrichment analysis for biological processes of upregulated and downregulated genes in hPSCs-derived GnRH neurons. **E** GO enrichment analysis for molecular function of upregulated genes. The GO cut-off criteria included q (adjusted *p* value) < 0.05. **F** Sub-heatmap of differentially expressed genes associated with neuronal development, ion channels and cell adhesion. **G** qPCR validation of genes showing differential expression in neurons derived from *RMST*-deleted hPSCs compared to control. Graphs show mean with ±SEM of 3–4 independent biological replicates and the data were analyzed using unpaired student t-test. Primer sequences are listed in Table S2. *p < 0.05, **p < 0.01, ***p < 0.001, ****p < 0.0001.

To understand transcriptional changes in GnRH neurons derived from WT and *RMST*-deleted hPSCs, we performed global gene expression analysis of GnRH neurons in three independent differentiation replicates. RNA was collected from neurons and analyzed by RNA sequencing (RNA-seq) to gain a comprehensive view of transcriptional differences between the neurons derived from WT and *RMST*-deleted cells (Fig. 4). Principle component analysis demonstrated good reproducibility of the experimental replicates (Fig. S3A). Comparison of controls and *RMST*-deleted neurons identified 1423 differentially expressed genes (DEGs) (adjusted *p*-value < 0.05), of which 717 were upregulated [fold change (FC) > 1.5] and 706 were downregulated [fold change (FC) < 0.5] (Fig. 4A, B). We performed Gene Ontology (GO) enrichment analysis (cut-off criteria of adjusted *p* value < 0.05) to identify biological processes/molecular functions associated with DEGs. The key biological process terms for upregulated genes showed their roles in nervous system development, cell-cell signaling, neurogenesis, generation of neurons, regulation of transport, neurons projection development, synaptic signaling, neurotransmitter transport and secretion (Fig. 4C). The key biological terms for downregulated genes showed their role in system development, cellular developmental process, cell differentiation, tissue development, movement of cell or subcellular component, cilium organization, cilium movement, and axoneme assembly (Fig. 4D). The molecular functions for the upregulated genes showed enrichment for GO terms including transporter activity, ion channel activity, cation channel activity, voltage-gated ion channel activity, potassium channel activity, voltage-gated potassium channel activity etc. (Fig. 4E), while the downregulated genes showed enrichment for GO terms including cytoskeletal protein binding, extracellular matrix structural constituent, tubulin binding, glycosaminoglycan binding, heparin binding, growth factors binding, and extracellular matrix structural constituent conferring tensile strength Fig. S3B). These results demonstrate that *RMST* deletion results in altered expression of genes involved in neurogenesis, neurotransmitter transport, synapse organization, cilium assembly and organization, epithelium development, and channel activity. Several of these dysregulated genes have previously been implicated to function in nervous system and hypothalamus development (*GAS1, GPR139, MAGED1, NNAT, NTRK2, POU6F2, BHMT, FGF13, TCEAL5, TMOD1, TNR*), ion channels proteins (*ASIC4, CACHD1, CACNA1B, CLCN5, GABRB2, GLRA2, KCNA2, KCNC1, KCND3, KCNH4, KCNJ3, KCNJ12, KCNJ13, SCN7A, SCN8A*) and cell adhesion proteins (*CDH8, DCHS2, PCDH7, PCDH10, PCDH15, PCDHA4*) (Fig. 4F). To validate the RNA-seq data, we performed qPCR analysis for several genes showing differential expression in neurons derived from *RMST*-deleted hPSCs compared to control (Fig. 4G). In conclusion, these results indicate that deletion in *RMST* caused altered expression of key genes involved in the development of hypothalamus and neuronal development, ion channels and cell adhesion proteins.

### Differential DNA methylation analysis

**Fig. 5:**
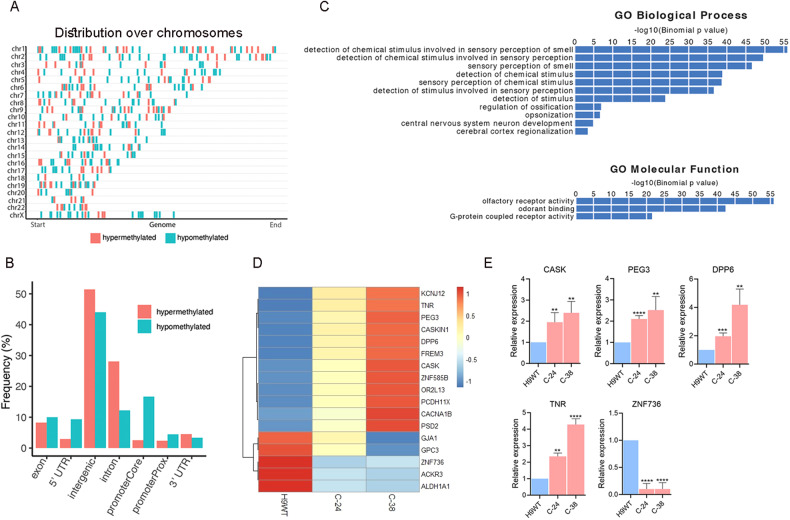
Differential DNA methylation analysis in *RMST*-deleted hypothalamic GnRH neurons. Distribution pattern of differential methylation regions (DMRs) across the genome (**A**) and distinct genomic element types (**B**): promoter, 5′-UTS, exon, intron, intergenic and 3’-UTR. (**C**) The DMR enrichment analyses using the GREAT annotation tool. (**D**) Sub-heatmap of RNA-seq results showing upregulation and downregulation of genes that colocalized with these DMRs. (**E**) qPCR validation of genes expression associated with DMRs. Graphs show mean ± SEM of 3–4 independent biological replicates and the data were analyzed using unpaired student t-test. Primer sequences are listed in Table S2. *p < 0.05, **p < 0.01, ***p < 0.001, ****p < 0.0001.

DNA methylation is an important epigenetic process that cells use to regulate gene expression and emerging evidence indicates intricate regulatory connections between lncRNA and DNA methylation [26]. To understand the effect of *RMST* deletion on DNA methylation, we performed DNA methylation analyses on WT and C-38 hPSCs-derived neurons using whole genome bisulfite sequencing. In total, 1759 differentially methylated regions (DMRs) were detected (*q* value < 0.05), out of which 669 regions were hypomethylated and 1090 were hypermethylated in C-38 hPSCs-derived neurons. These regions are distributed across the genome (Fig. 5A) in 5′ UTR, promoter regions, exons, introns and 3′ UTR (Fig. 5B). For hypermethylated DMRs, 2.5% occurred at the core promoter, 2.4% at the proximal promoter, 4.5% at the 3′untranslated regions (3’UTR), 2.9% at the 5′UTR, 8.2% at exons, 28% at introns, and 51.4% in intergenic regions. Whereas 16.7% of hypomethylated DMRs occurred at the core promoter, 4.4% at the proximal promoter region, 3.3% at the 3′UTR, 9.3% at the 5’UTR, 10% at exons, 12.2% at introns, and 44% at intergenic regions (Fig. 5B).

We checked whether DMRs associated with *RMST* loss were linked to specific biological and molecular processes. The DMR enrichment analyses were performed using the GREAT annotation tool for the hypomethylated and hypermethylated regions, independently. Our findings revealed that hypermethylated targets following *RMST* deletion are enriched for diverse biological processes, including detection of chemical stimulus involved in sensory perception of smell, sensory perception of smell, regulation of ossification, opsonization, central nervous system neuron development (Fig. 5C). The molecular processes affected by these DMRs were olfactory receptor activity, odorant binding, and G protein-coupled receptor activity (Fig. 5C). The hypomethylated targets following *RMST* deletion are enriched only for anterior/posterior pattern specification (Fig. S3C). Our RNA-seq results show that several of these DMRs corresponded with the upregulation and downregulation expression of colocalized genes (Fig. 5D), which were confirmed by qPCR for several examples (Fig. 5E). In conclusion, these results demonstrate that *RMST* lncRNA deletion altered genomic DNA methylation in hPSCs-derived neurons.

## Discussion

Kallmann Syndrome (KS) is a rare genetic disorder that combines congenital hypogonadotropic hypogonadism with olfactory dysfunction. Mutations in more than 30 genes affecting GnRH neurons development/migration in KS patients have been identified [3], however, our understanding of the genetic basis of the syndrome remains incomplete. Moreover, the disruption of GnRH neuron neurogenesis has received much less attention than GnRH neuronal migration in the context of KS or CHH. Human pluripotent stem cells (hPSCs) which can self-renew indefinitely while maintaining the potential to become any cell type of the human body [27, 28] provide opportunity to model GnRH neurons in vitro and understand the biological mechanism regulating the differentiation of these neurons. Furthermore, the use of CRISPR/Cas9 genome editing in combination with hPSCs would help to investigate in vitro the function of KS candidate genes in GnRH neurons development. Here we wanted to understand the function of *RMST* lncRNA in GnRH neurons which has been previously shown to regulate neurogenesis and a balanced translocation in *RMST* also caused Kallmann syndrome [14, 22], however, there is no in vitro model to study the function of *RMST* in GnRH neurons. Our results show that *RMST* is essential for the proper functioning of GnRH neurons and deletion in *RMST* caused hyperexcitability and increased calcium influx activity. These cells could provide a useful platform to further understand the mechanism of *RMST* in the progression of Kallmann syndrome.

Recent studies indicate that lncRNAs are involved in homeostasis and function of the mammalian brain [15, 16] and many of these lncRNAs are expressed in the embryonic and adult mammalian brain in a highly patterned and specific manner [29, 30]. LncRNAs play an essential role in regulating gene expression at different level including chromatin organization, transcription control and post-transcription regulation [31]. Our results show that *RMST* deletion does not affect the differentiation potential of stem cells into GnRH neurons. The GnRH staining appeared in punctuate structures, indicating GnRH prepropeptide processing into mature decapeptide and packaging into secretory vesicles. Bulk RNA-seq analysis revealed that *RMST* deletion results in the dysregulation of many genes in GnRH neurons that have essential functions in hypothalamus and neuronal development like *POU6F2, TNR, TCEAL5, RIT2, GPR139, NNAT, PEG3, TOX2, CASK, GAS1, GLRA2, BHMT, MITF, RDH10 and PAX7*. POU Domain, Class 6, Transcription Factor 2 (POU6F2) mRNA was highly expressed in GnRH neurons and upregulated in *RMST-*deleted cells. Most POU family members function as transcriptional regulators, controlling cell type-specific differentiation [32], and modulate the development, expression, and function of GnRH neurons [33–35]. Moreover, DNA methylation analysis revealed differential methylation regions in *RMST*-deleted neurons and some of these DMRs were associated with biological and molecular processes like sensory perception of smell, odorant binding and olfactory receptor activity. Dysfunction of the olfactory system is a key feature of Kallmann syndrome and distinguishes it from other forms of CHH. Several of these DMRs were also regulating the expression of colocalized genes. For example, Tenascin-R (*TNR*) was hypomethylated and upregulated in *RMST*-deleted neurons. TNR is key element of perineuronal nets which are localized around neurons during development and are specialized forms of neural extracellular matrix with neuroprotective and plasticity-regulating roles [36]. The atypical chemokine receptor 3 (*ACKR3*) was hypermethylated and downregulated in *RMST*-deleted neurons. ACKR3 is expressed in brain [37] and plays a role in interneurons migration [38, 39].

*RMST* deletion increased calcium influx activity and action potential frequency in hPSCs-derived GnRH neurons, suggesting a hyperexcitability phenotype. However, alternative explanations, such as the promotion of maturation, cannot be ruled out. Neuronal excitability is dependent on electrochemical gradients that drive the passage of ions across the membrane through ion channels. Our RNA-seq data show that *RMST* deletion results in the altered expression of several ion channels, the pore-forming membrane proteins involved in the regulation of membrane potential and for the initiation and propagation of action potentials [40, 41]. The mRNA expression of several genes encoding potassium channels (*KCNA2, KCNA3, KCNC1, KCN1D1, KCND3, KCNH4, KCNH5, KCNJ3, KCNJ9, KCNJ12*) and calcium voltage-gated channels (*CACNA1I, CACNA1B, CACNB1, CACNG5, CACNG7*) were dysregulated in *RMST-* deleted neurons. In addition, mRNA expression of genes encoding sodium voltage-gated channels (*SCN3B, SCN7A, SCN8A*) were upregulated in *RMST*-deleted neurons. Functional analysis using gene ontology revealed the association of many upregulated genes in *RMST*-deleted neurons with the ion channels activity, proposing the maturation as well as hyperactivity of neurons. Further validation of our transcriptomic findings with functional assays, such as standard patch-clamp recordings for the current density and resting membrane potential is needed to establish a direct link between gene expression changes and neuronal function. Incorporating these measurements would indeed strengthen the conclusions drawn from our study.

In conclusion, the results presented here show that *RMST* lncRNA deleted hPSCs could be efficiently differentiated into GnRH neurons. The resulting neurons were functionally hyperactive with increased calcium influx activity. Moreover, *RMST* deletion resulted in the dysregulation of many genes involved in neuronal development, ion channels and cell adhesion. The in vitro generation of these *RMST*-deleted GnRH neurons provides an excellent cell-based model to understand the molecular mechanism of *RMST* in Kallmann syndrome and hypothalamic neuronal development.

## Materials and methods

### Ethics statement

In the present study, pluripotent H9 ESC (WA09, WiCell Research Institute) was used. All work was reviewed and approved by the institutional review board in Hamad Bin Khalifa University (QBRI-IRB 2018-024).

### Cell culture

H9 wild-type and *RMST*-deleted cells were cultured in the presence of humidified atmosphere and 5% CO_2_ in a 37 °C incubator and maintained under feeder-free conditions in mTeSR1 medium (Stem cell Technologies, Vancouver) on Matrigel (1:80, BD Biosciences) coated culture plates. The culture medium was changed daily, and colonies were passaged as small clumps using Gentle cell dissociation reagent (Stem cell technologies). The cells were checked for mycoplasma using the primers listed in Table S2.

### *RMST* gene deletion using CRISPR-Cas9

For *RMST* deletion, the guide RNA (gRNA) sequences targeting upstream of exon 3 and downstream of exon 8 (listed in table S1) were selected using CRISPR-Cas9 gRNA design tool (Integrated DNA technologies). Single guide RNA (sgRNA) was synthesized using EnGen sgRNA Synthesis Kit (NEB, E3322) according to the manufacturer’s instructions and purified using Monarch RNA cleanup kit (NEB, T2040). Nucleofection was carried out using the Amaxa nulceofection system (P3 primary cell 4D-nucleofector kit, Cat No. V4XP-3032) according to the manufacturer’s instructions. Briefly, ribonucleoproteins (RNP) complex were generated by mixing 1 μg of each sgRNA with 2 μM of EnGen SpyCas9 NLS (NEB, M0646) at room temperature for 15–20 min. Approximately, 3 × 10^5^ hPSCs were electroporated using CB150 nucleofection program and plated onto Matrigel-coated plates. After 48 h, the cells were detached using TrypLE (Cat No. 12604013) and plated as a single cell on Matrigel-coated plates for 10–15 days to make colonies. Genomic DNA (gDNA) was extracted using quick extract genomic DNA extraction buffer (epicenter). The cleavage efficiency of sgRNA was evaluated using T7 endonuclease I (NEB #E3321) cleavage assay. The region of *RMST* targeted by sgRNAs was amplified with specific primers (listed in table S1) using PCR-Master mix (Thermo Fisher Scientific) and deletion was confirmed by sanger sequencing of the PCR products.

### Differentiation of hPSCs into GnRH neurons

hPSCs were differentiated into GnRH neurons following a published protocol [23, 24] with minor modifications. Briefly, hPSCs colonies were dissociated into single cells using TrypLE (Thermo Fischer Scientific) and plated onto matrigel-coated (1:80) plates in mTeSR1 medium containing 10 μM Y-276321 (ROCK inhibitor). The next day, cells were 80–90% confluent and differentiation was initiated by changing medium to Neurobasal medium (DMEM/F12, Neurobasal, 1X B-27 minus vitamin A, 1X N2 supplement, 1X l-Glutamine, 1X Non-essential amino acids (NEAA), 50 μM 2-mercapto-ethanol, 0.2X Penicillin/streptomycin) supplemented with dual SMAD inhibitors 10 μM SB431542 (TGF-β inhibitor) and 2 μM Dorsomorphin (BMP inhibitor) for 12 days. During neural induction, cells were split at days 8 and 14 using TrypLE and plated onto Matrigel-coated plates in Neurobasal media containing 5 μM Rock inhibitor. On day 12, dual SMAD inhibitors were withdrawn, and cells were cultured until day 20 in Neurobasal medium supplemented with 20 ng/ml FGF8. On day 20, cells were dissociated with TrypLE and replated at a dilution of 1:5 in Neurobasal medium containing FGF8 and 5 μM ROCK inhibitor. On day 21, the Neurobasal medium was supplemented with 20 ng/ml FGF8 and 20 μM Notch inhibitor DAPT (2 *S*)-*N*-[(3,5-Difluorophenyl) acetyl]-L-alanyl-2-phenyl] glycine 1,1-dimethylethyl ester, for one week to differentiate into GnRH neurons.

### Immunostaining

Cells were washed with 1X PBS and fixed with 4% paraformaldehyde for 15–20 min at room temperature. The fixed cells were washed three times with PBS and permeabilized with 0.2% Triton X-100 (Sigma-Aldrich) in PBS for 30 min and blocked in PBST (PBS supplemented with 0.2% tween-20) containing 3% bovine serum albumin (BSA) for 2–3 h. The cells were incubated with primary antibodies overnight at 4 °C. Primary antibodies consisted of SOX2 (Rabbit, 1:200, Invitrogen: MA1-014), PAX6 (Mouse, 1:100, Abcam: ab78545), OTX2 (Goat, 1:300, R&D: AF1979), FOXG1 (Rabbit, 1:200, Abcam: ab18259), Nestin (Mouse, 1:100, Invitrogen: MA1110), MAP2 (chicken, 1:500, Abcam: ab5392), Ki67 (Rabbit, 1:100, Abcam: ab833), GnRH (Rabbit, 1:200, Sigma: HPA027532). Next day, the cells were washed and incubated with the secondary antibodies (1:1000) in PBST containing 3% BSA for 1 h at room temperature. Secondary antibodies were conjugated with Alexa Flour 488 and Alexa Flour 555 (Thermo Fischer Scientific). Nuclei were stained with DAPI (Thermo Fischer Scientific) for 10 min. Cells were washed three times with PBST and images were taken using the inverted fluorescence microscope (Olympus IX 53).

### RNA extraction, real-time PCR, and library preparation

For RNA extraction, the cells were lysed in TRIzol (Thermo Fischer Scientific), and total RNA was purified using Direct-zol RNA MiniPrep Extraction Kit (Zymo Research) according to the manufacturer’s instructions. Complementary DNA (cDNA) were synthesized from 0.5 µg of RNA using RevertAid First Strand cDNA Synthesis kit (Thermo Fischer Scientific). Quantitative PCR (qPCR) was performed using Syber Green PCR Master Mix (Applied Biosystems) and amplification was detected using Quant Studio 7 system (Applied Biosystems). Gene expression was normalized to *GAPDH*. The primer details are listed in Table S2.

For library preparation, total RNA with an RNA integrity number (RIN) above 8 was used as input using TruSeq Stranded mRNA kit (Cat #: 20020594) from Illumina following the manufacturer’s instructions. Briefly, 0.5 µg of total RNA was used to capture mRNA molecules using poly-T oligo-attached magnetic beads. The mRNA was fragmented, and cDNA was generated using random priming during first and second-strand synthesis. Barcoded DNA adapters were ligated to both ends of DNA, and then amplified. The quality of library generated was checked on an Agilent 2100 Bioanalyzer system and quantified using a Qubit system. Libraries that pass quality control were pooled, clustered on a cBot platform, and sequenced on an Illumina HiSeq 4000 at a minimum of 20 million paired-end reads (2×75 bp) per sample.

### RNA-Seq data analysis

For RNA-seq analysis, the low-quality reads were discarded, and adapter sequences were trimmed using Cutadapt with default parameters. The high-quality paired-end reads from each sample were aligned to the Human reference genome (GRCh38/hg38) using STAR version 2.7.10 [42] and transcript counting was carried out using Subread:featureCounts version 2.0.1 [43]. All gene-level transcript counts were imported in R and differential expression analysis was performed with DESeq2 [44]. Genes with adjusted *p* values < 0.05 and absolute fold changes > 1.5 were considered as differentially expressed. The volcano plot and heatmap were created using the ggplot2 and Pheatmap R-libraries respectively. Gene Ontology (GO) enrichment analysis was performed using ShinyGO package [45].

### Whole genome bisulfite sequencing and differential DNA methylation analysis

Genomic DNA was isolated from two biological replicates of wild type and *RMST*-deleted C-38 derived GnRH neurons and bisulfite converted using the EZ DNA Methylation-Gold Kit (Zymo Research). Next, Whole Genome Bisulfite (WGBS) libraries were prepared using the Accel-NGS Methyl-Seq DNA Library Kit and sequenced (150 bp paired-end) on a NovaSeq 6000 at the genomics core facility of Weill-Cornell Medical College – Qatar with ~ 20x coverage per library. The quality of the generated FASTQ files were checked using FastQC and trimmed using Trim Galore to remove any adapter contamination (http://www.bioinformatics.babraham.ac.uk/projects/trim_galore/). In total, 10 bp each were removed from the 5’ and 3’ position of Read1 while 15 bp and 10 bp were removed from the 5’ and 3’ position of Read2, respectively.

Using Bismark, the trimmed reads were aligned against the human reference genome (GRCh38/hg38) [46]. The mapping efficiency of the sequenced H9WT and KO libraries was ~75%. The output-aligned reads (BAM) were coordinately sorted using samtools followed by deduplication. The deduplicated reads were query-sorted and the M-Bias report was generated to check and remove any bases with methylation bias at the 5′ and 3′ ends. For all WGBS libraries, no methylation bias was observed and therefore no additional bases were removed. Next, CpG methylation extraction was performed using the “bismark_methylation_extractor” function and coverage files were generated using the “coverage2cytosine” function along with the --merge_CpG option. The output coverage files were later used to perform differential methylation analysis using the R package DMRseq [47]. DMRseq works on a permutation-based approach for performing differential methylation analysis and can detect DMRs accurately even from a small sample size of two per group. Using DMRseq, differential methylation analysis was performed and significant DMRs with q-value (FDR adjusted *p*-value) < 0.05 were filtered. Subsequent gene ontology enrichment analysis was performed using the Genomic Regions Enrichment of Annotations Tool (GREAT) [48].

### Electrophysiology

Recordings of action potentials (APs) using the conventional whole-cell conFigureuration of the patch-clamp technique were carried out, as previously reported [49]. The neurons were cultured on coverslips and perfused with the normal Tyrode’s (NT) bath solution (mM): 143 NaCl, 5.4 KCl, 0.33 NaHPO_4_, 0.5 MgCl_2_, 5 HEPES, 2 CaCl_2_, and 11 glucose; pH 7.4 adjusted with NaOH. The patch pipettes were pulled from borosilicate capillary tubes (A-M systems, WA, USA) using a PC-10 puller (Narishige, Tokyo, Japan) and filled with an internal solution (mM): 130 K-gluconate, 3 KCl, 2 MgCl_2_, 10 HEPES, 5 Na2ATP, 0.5 Na_2_GTP, 0.2 EGTA; pH 7.3 adjusted with KOH. Data acquisition, voltage control, and analysis were accomplished using the HEKA Patchmaster software. For recording of action potentials, a current-clamp mode was used under the conventional whole-cell conFigureuration with a series of current steps from −20 to +60 pA for 500 ms. Signals were low-pass filtered with a cut-off frequency of 5 kHz and sampled at 10 kHz.

### Single-cell calcium imaging

Neurons cultured on coverslips were incubated with 3 µM Fura-2 AM (Thermo Fisher Scientific) for 30 min at room temperature in a normal Tyrode’s buffer solution [49]. Calcium imaging experiments were conducted using a spectrofluorometric system based on a monochromator (Photon Technology International, Lawrenceville, NJ) with an Evolve 512 camera (Teledyne Photometrics, AZ, USA). Dual excitation and emission were at 340/380 and 510 nm, respectively. Data acquisition was performed using the EasyRatioPro software. To induce calcium influx through voltage-gated calcium channels (VGCCs), a solution containing 50 mM KCl was applied to depolarize the membrane potential. Regions of interest (ROI) were assigned by highlighting the perimeter of the cell using the software. Mean fluorescence intensity was recorded within the ROIs. Changes in fluorescence intensity were analyzed after background subtraction using ImageJ software (National Institutes of Health, Bethesda, MD).

### Karyotype analysis

The hPSCs at 70–80% confluency were treated with 100 ng/ml KaryoMax colcemid (Thermo Fisher Scientific) for 3 h to arrest the cells at metaphase. The cells were then detached using TrypLE and treated with hypotonic solution (0.75 M KCL, Thermo Fisher Scientific) for 20 min at 37 °C. The cells were fixed in methanol: glacial acetic acid (3:1) and karyotype analysis was carried out in Universitätsklinikum, Institut für Humangenetik (Germany) using standard protocols for G-banding.

### Statistical analysis

Data analysis was performed using GraphPad Prism 9 (GraphPad Software, San Diego, CA, USA). Data are means ± standard error of the mean (SEM). An unpaired two-tailed t-test was used to compare gene expression and estimate the statistical significance between two samples. Data from whole-cell patch-clamp recording and single-cell calcium imaging were analyzed using One-way ANOVA with Tukey’s multiple comparisons. Probabilities of *p* < 0.05 were considered significant.

### Supplementary information

Supplementary material

Figure S1

Figure S2

Figure S3

## Data Availability

The RNA-seq datasets generated and used in the present study are publicly available on the Zenodo repository: https://zenodo.org/records/12514420. Further information and requests for resources and reagents should be directed to the corresponding author (E-mail: LStanton@hbku.edu.qa).
