# Supplementary material for: Deletion in RMST lncRNA impairs hypothalamic neuronal development in a human stem cell-based model of Kallmann Syndrome
Source: Cell Death Discov. 2024 Jul 19;10:330. doi: 10.1038/s41420-024-02074-4 (PMC11271498; doi:10.1038/s41420-024-02074-4)
Supplement: Supplementary file 1 — Supplementary material [file 41420_2024_2074_MOESM1_ESM.docx]

Figure S1: (A) Agarose gel electrophoresis of genomic DNA for the validation of deletion in *RMST* gene in single cell derived clones. Forward primers (FP1 and FP2) and reverse primers (RP2) were used to screen the clones for heterozygous and homozygous deletion. FP1 primer binds upstream of exon 3, FP2 and RP2 bind upstream and downstream of exon 8 respectively. The binding site for FP2 is lost in *RMST*-deleted clones. In clones having homozygous deletion, the primer pair (FP2 and RP2) yields no PCR amplification and primer pair (FP1 and RP2) results in PCR amplification. (B) Karyotyping analysis of RMST-deleted clones (C-24 and C-38) in hPSCs using GTG stained metaphase preparations.

Figure S2: (A) qPCR of progenitor markers in hPSC derived neural progenitor cells (NPCs) at day 20 of differentiation. No differences in the expression of progenitor markers were observed in control and *RMST*-deleted hPSC-derived NPCs. Graphs show mean ±SEM of 3-4 independent biological replicates and the data were analyzed using unpaired student t-test. Primer sequences are listed in table S2. (B) Immunostaining of mature GnRH neurons showing the expression of ki67 (green) on day 28 of differentiation. Cell

nuclei were stained with DAPI (blue). Scale, 100 µm. **p* < 0.05, ** *p* < 0.01, *** *p* < 0.001, **** *p* < 0.0001.

Figure S3: (A) RNA-seq analysis of hPSC-derived hypothalamic GnRH neurons. (A) Principal component analysis (PCA) plot of RNA-seq data. Each data point represents an independent RNA-Seq experiment. Sample groups are indicated by different colors as indicated in the legend. (B) Gene ontology (GO) enrichment analysis for molecular function of downregulated genes. The results showed enrichment for GO terms including calcium ion binding, glycosaminoglycans binding, heparin binding, growth factors binding and extracellular matrix structural constituent conferring tensile strength. The GO cut-off criteria included q (adjusted p value) < 0.05. (C) The DMR enrichment analysis for hypomethylated targets using the GREAT annotation tool shows enrichment for anterior/posterior pattern specification.

Table S1: Sequence of guide RNAs targeting RMST gene and genomic DNA primers.

| **Primer** | **Forward primer 5ˈ- 3ˈ** |
| --- | --- |
| FP1 | GCTTCTCCTTCTGAACAGAGCGCTTTGCTC |
| RP1 | GTAGACACAATCGCCACTTATCTCACAATC |
| FP2 | GCTGTAGGAGAAGCTAACGAATTATTTGCAAT |
| RP2 | GTAGAGAAGGATGTTATCAGTAGTTCAGCAC |
| gRNA1 | GACAGGACCATGTTACACTC |
| gRNA2 | TGTCAAGGTAGAAAAGTCCA |

Table S2: Sequence of RT-PCR and qPCR primers

| **Gene** | **Forward primer 5ˈ- 3ˈ** | **Reverse primer 5ˈ- 3ˈ** |
| --- | --- | --- |
| OCT4 | GACAGGGGGAGGGGAGGAGCTAGG | CTTCCCTCCAACCAGTTGCCCCAAAC |
| NAN | CATGAGTGTGGATCCAGCTTG | CCTGAATAAGCAGATCCATGG |
| KLF4 | CAGTCCCGGGGATTTGTAGC | GAAGAAGGTGGGGTGAGCAT |
| c-MYC | TTCTGTGGAAAAGAGGCAGG | TGCGTAGTTGTGCTGATGTG |
| GnRH1 | GAGCTCTGGAAAGTCTGATTGA | GCAACTTGGTGTAAGGATTTCTG |
| KISS1R | ACTTCTACATCGCCAACCTG | GTTGACGAACTTGCACATGAA |
| SOX2 | GAACCATCTCTGTGGTCTTGTT | TTACCAACGGTGTCAACCTG |
| FOXG1 | CTTTACCCTGTGTTTATTTC | GTGCATTATAGTCACTTCTA |
| OTX2 | CTCACTCGCCACATCTACTTTG | GGTTTGGAGCAGTGGAACTTA |
| GAD1 | CTTCACCTCAGAACAGAGTCAC | CCAGCAGTTGCATTGACATAAA |
| NNAT | TTGCGAGAAGTGAGGTGTTC | CACAGGAGCACCTGATGATAC |
| RET | GGAGATGGCAAAGGGATCA | TCTTGGGTCTCCACAACATC |
| CDH9 | TAGGCAAGCTTCACACTGAC | GCAGCATGAATGTCTCCTGTA |
| CASK | AGGGTCCCTTCAGTGTTGTA | GGCTTCCCGCTTTAGATCTTC |
| DPP6 | TCCGCAGAGGAATTGGAAAG | ATTATCTTCCGCTGGTGTCAG |
| GLRA2 | GTGCAAGTTGCTGAAGGATTG | GCAGGTAAACTTTCCAGTGTTG |
| TCEAL5 | CAGTATCGGTGCAGGAAAGG | TCTGTACTTCCCTCATCCTCTG |
| ORAI1 | TTCCAGTGCTTTGGCCTTAC | AATCCTCTTCCCTCCATGCT |
| PAX7 | ACAGCTTCTCCAGCTACTCT | CCAAGATGCTCATCACCTGAG |
| FOXP2 | CAGGCTATCATGGAGTCATCTG | ACGTACTGCATTCTTCCAAGT |
| ZNF736 | GTCTCCTTGGGTCTTGCTATC | GTCATGATCCGGCAATATCTCT |
| PEG3 | CATCGAGCTCTTGGTCCTTG | GTTGTCGTCTTCTGGTTGGT |
| POU6F2 | CTGGAGGAGATCCGAGAATTTG | CTTCTCAGGATGGTGTGTCTG |
| KCNJ12 | CTGACCGAGGATGTTCTAGTG | CGGCTCCTCTTGAGTTCTATC |
| GAPDH | ACGACCACTTTGTCAAGCTCATTTC | GCAGTGAGGGTCTCTCTCTTCCTCT |
| RMST RT-PCR | TCTGCACTTTCCTGGTTCTATG | GTCTAAGGCAGGTCTCCAGTAA |
