# Supplementary figures and images for: Deletion in RMST lncRNA impairs hypothalamic neuronal development in a human stem cell-based model of Kallmann Syndrome
Source: Cell Death Discov. 2024 Jul 19;10:330. doi: 10.1038/s41420-024-02074-4 (PMC11271498; doi:10.1038/s41420-024-02074-4)

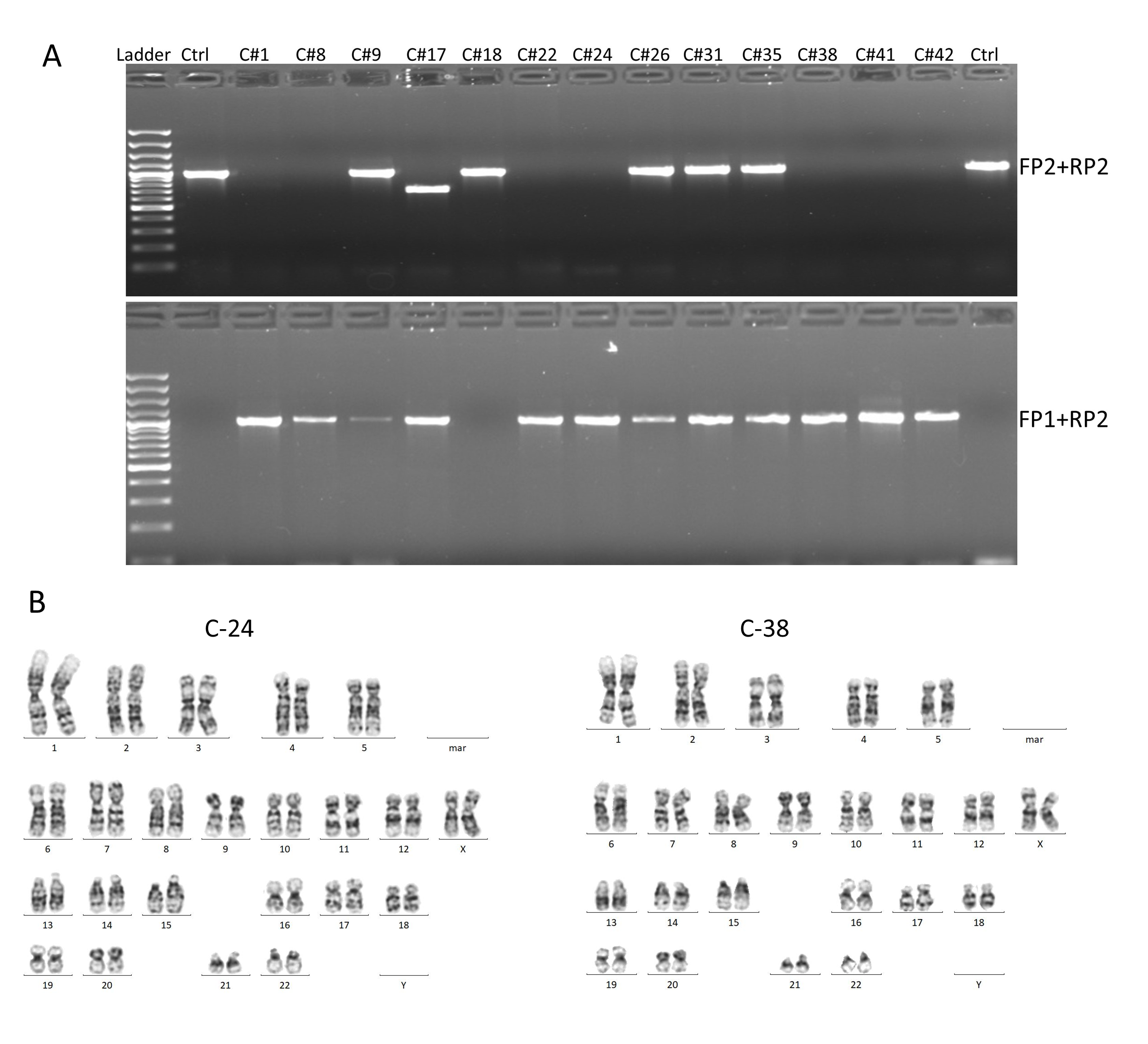

Supplement: Supplementary file 2 — Figure S1 [file 41420_2024_2074_MOESM2_ESM.tif]

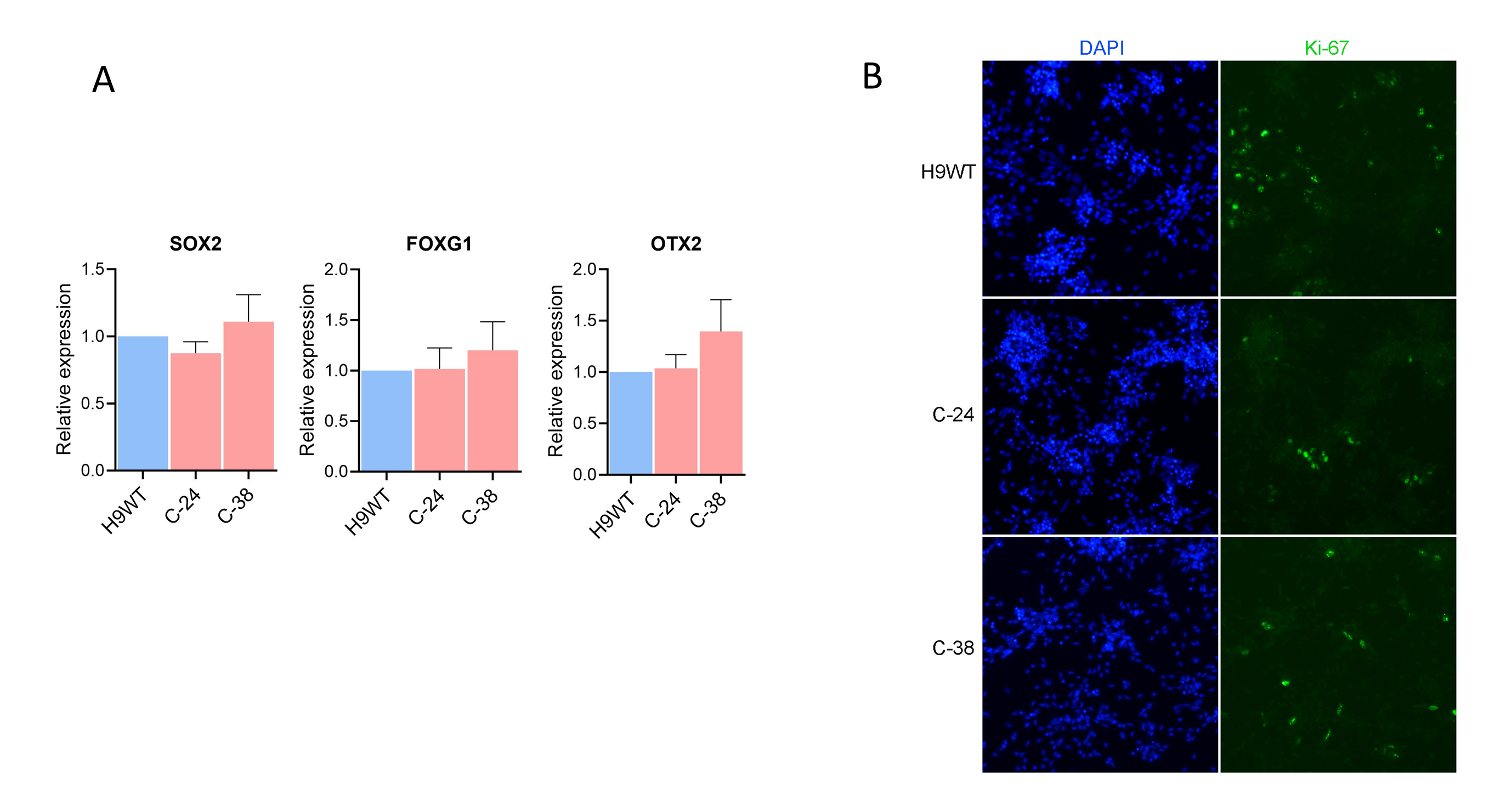

Supplement: Supplementary file 3 — Figure S2 [file 41420_2024_2074_MOESM3_ESM.tif]

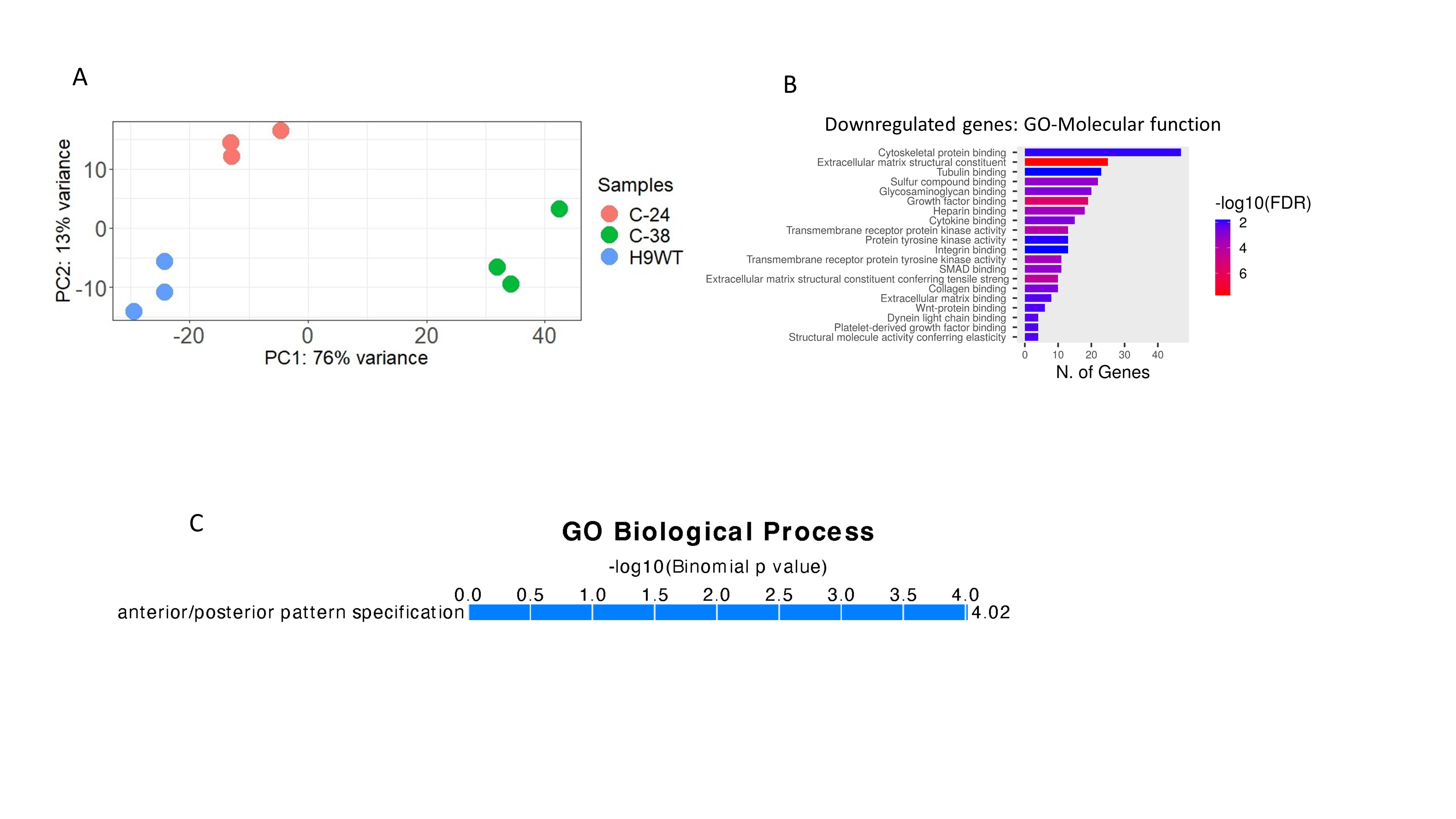

Supplement: Supplementary file 4 — Figure S3 [file 41420_2024_2074_MOESM4_ESM.tif]
